# The Capabilities Approach: Fostering contexts for enhancing mental health and wellbeing across the globe

**DOI:** 10.1186/s12992-016-0150-3

**Published:** 2016-05-05

**Authors:** Ross G. White, Maria Grazia Imperiale, Em Perera

**Affiliations:** Institute of Health and Well-being, University of Glasgow, 1st Floor, Admin Building, Gartnavel Royal Hospital, 1055 Great Western Road, Glasgow, G12 0XH UK; School of Education, The University of Glasgow, Glasgow, UK; Enhance Living, Kent, UK

**Keywords:** Capabilities approach, Recovery approach, Wellbeing, Global mental health

## Abstract

Concerted efforts have been made in recent years to achieve equity and equality in mental health for all people across the globe. This has led to the emergence of Global Mental Health as an area of study and practice. The momentum that this has created has contributed to the development, implementation and evaluation of services for priority mental disorders in many low- and middle-income countries.

This paper discusses two related issues that may be serving to limit the success of mental health initiatives across the globe, and proposes potential solutions to these issues. First, there has been a lack of sophistication in determining what constitutes a ‘good outcome’ for people experiencing mental health difficulties. Even though health is defined and understood as a state of ‘wellbeing’ and not merely an absence of illness, mental health interventions tend to narrowly focus on reducing symptoms of mental illness. The need to also focus more broadly on enhancing *subjective wellbeing* is highlighted. The second limitation relates to the lack of an overarching theoretical framework guiding efforts to reduce inequalities and inequities in mental health across the globe. This paper discusses the potential impact that the Capabilities Approach (CA) could have for addressing both of these issues. As a framework for human development, the CA places emphasis on promoting wellbeing through enabling people to realise their capabilities and engage in behaviours that they subjectively value.

The utilization of the CA to guide the development and implementation of mental health interventions can help Global Mental Health initiatives to identify sources of social inequality and structural violence that may impede freedom and individuals’ opportunities to realise their capabilities.

## Background

The Global Burden of Disease Study 2010 indicated that mental disorders pose a striking and growing challenge for health systems in both developed and developing regions of the world [1]. Approximately 80 % of the world’s population live in low- or middle-income countries (LMIC) [2]. LMIC tend to allocate less than 2 % of the health budget to mental health, compared to as high as 10 % in high-income countries [3]. It is claimed that up to 90 % of people living in some low-income countries who require treatment for mental disorders do not receive it [4]. This has been referred to as the ‘treatment gap’ – the gap between the demand for treatment and what is available on the ground. Over the last 15 years, Global Mental Health (GMH) has developed as an area of research and practice that places a priority on improving mental health and achieving equity in mental health for all people worldwide [5]. Whilst GMH initiatives such as mhGAP [6, 7] have done much to build capacity for delivering and evaluating interventions for mental health difficulties in many LMIC, contention exists regarding the comparative merits and demerits of these endeavours (see [8–10]). Concerns have been raised about a lack of theoretical coherence in efforts to build capacity for mental health services across the globe. Prof Joop de Jong (speaking at the ‘*Global Mental Health: Bridging the Perspectives of Cultural Psychiatry and Public Health*’ meeting at McGill University in 2012) pointed out that ‘the function of GMH remains unclear since it is lacking a (meta-) theory to guide its action. Without such theory, there is an impediment to developing testable models related to cooperation, and to solving major preoccupations related to access to care or stigma’ [11]. In addition, we suggest that efforts to build capacity for mental health services are being limited by a lack of sophistication in determining what actually constitutes a 'good outcome' for people in the particular contexts in which they are living their lives. Without this, GMH may fall victim to the futility of what George Santayana refers to as ‘redoubling your efforts when you have forgotten your aim’ [12].

Theorists have proposed that *mental illness* and *mental health* exist on distinct continua [13]. Consistent with this approach, the World Health Organization has sought to define ‘mental health’ in its own right rather than merely describing it as the absence of mental illness. For example, the *Mental Health Action Plan 2013–2020* [14], which aims to extend mental health services and reduce inefficiencies in service provision across the globe, conceptualizes mental health ‘as a state of *wellbeing* in which the individual realizes his or her own abilities, can cope with the normal stresses of life, can work productively and fruitfully, and is able to make a contribution to his or her community’. The term ‘subjective wellbeing’ (SWB) has been used to describe a state of satisfaction with life and emotional equilibrium [15]. To guard against the risk that SWB places too much emphasis on the gratification of transient hedonistic needs (see [16]), it is suggested that conceptualisations of ‘wellbeing’ should incorporate the Aristotelian concept of ‘eudaimonia’ (from 'daimon' i.e. the true nature) [17]. Eudaimonia can be understood as a sense of experience derived from an ethical way of life, mental balance and wisdom. High levels of eudaimonia have been linked to people having an increased purpose in life, and greater levels of social integration, personal growth, social contribution, and autonomy [18]. ‘Flourishing’ has been introduced as a term to describe individuals with high levels of emotional, psychological and social wellbeing, and the term ‘languishing’ is used to describe individuals who are experiencing low levels of emotional, psychological, and social wellbeing [18]. The *Mental Health Continuum - Short Form* (MHC-SF) [19], which was originally developed in the US, is a measure of ‘flourishing’ that consists of 14 items; 3 items assessing emotional wellbeing, 6 items assessing psychological wellbeing, and 5 items assessing social wellbeing. Recent research from the Netherlands has indicated that a psychotherapy intervention can serve to increase levels of flourishing [20]. However, further research is required to investigate the potential validity of the MHC-SF across different cultural contexts.

Research conducted in Zambia and India has highlighted that conceptualizations of wellbeing are influenced by the particular social, political and cultural contexts in which people live [16]. As such, it has been suggested that ‘forms of local understandings of wellbeing need to be explored not assumed, and qualitative methods are vital to achieve this’ [21] (p.2720). White and colleagues [16] introduced the term ‘Inner Wellbeing’ (IWB) to distinguish culturally-informed notions of ‘wellbeing’ from more standardized conceptualisations of SWB that are largely defined in terms of ‘internal psychology’ (i.e. intrapsychic phenomena). Seven separate domains were identified as being important for measuring wellbeing for participants recruited from India and Zambia - economic confidence, agency and participation, social connections, close relationships, physical and mental health, competence and self-worth, values and meaning [16]. Although measuring SWB across different cultural settings is not without its challenges, a recent systematic review has indicated that SWB is a cross-culturally valid concept and that meaningful comparisons across these settings are possible [22].

In spite of the burgeoning research being conducted into SWB, the vast majority of studies evaluating the efficacy of mental health interventions continue to narrowly define ‘good outcome’ according to a reduction in symptoms of mental illness and/or a reduction in levels of disability. It has been suggested that ‘the study of subjective wellbeing must take deeper institutional roots to begin to foster research and its application toward understanding how to add more health to human life expectancy’ [18] (p.7). Indeed, efforts to identify the social, economic and political conditions that promote subjective wellbeing of populations may help augment efforts to reduce the burden associated with mental disorders [23]. Although we recognise that initiatives aimed at reducing symptoms of mental illness will continue to play an important role, moving forward we propose that initiatives aimed at supporting individuals who are experiencing mental health difficulties could benefit from including contextually sensitive approaches aimed at enhancing SWB irrespective of their diagnosis, symptom levels, and/or location on the globe.

Over the last 20 years the *Recovery Approach (RA)* [24] has been instrumental in highlighting that understanding about prognosis related to mental health difficulties needs to move beyond a narrow focus on symptom remission alone, to a broader interest in individuals’ subjective appraisals about their experiences and the meaning that they attribute to these experiences. From a RA perspective, ‘personal recovery’ can be defined as a ‘unique process of changing one’s attitudes, values, feelings, goals, skills, and/or roles. It is a way of living a satisfying, hopeful, and contributive life - even within the limitations caused by illness. ‘Personal recovery’ involves the ‘development of new meaning and purpose in one’s life as one grows beyond the catastrophic effects of mental illness [24] (p.17). This can be contrasted with ‘clinical recovery’ that emphasises ‘the invariant importance of symptomatology, social functioning, relapse prevention and risk management’ [25] (p.1). It is claimed that the emphasis that the RA places on exploring individuals’ own goals and strengths is advantageous for promoting subjective wellbeing [25].

Although the RA has done much to highlight the importance of the subjective meaning that individuals may take from their mental health difficulties, a number of concerns have been raised about potential limitations associated with the approach. For example, it has been suggested that aspects of the RA have been misappropriated, misrepresented or abused in the commissioning, design and implementation of services [26]. We propose that a ‘recovery gap’ exists in mental health services i.e. there is a gap between the types of outcomes that individuals with a lived experience of mental health difficulties want to prioritise (e.g. gaining hope, a sense that life has meaning, a feeling of empowerment) and the willingness and/or capacity of services to deliver on these outcomes. Furthermore, we propose that the RA is limited in the extent to which it can address macro-level factors that may be limiting opportunities for citizenship and/or impacting on subjective wellbeing (e.g. sources of social injustice such as inequality, stigma and discrimination).

The *Capabilities Approach* (CA) [27] is an approach that complements the ethos of the RA and also provides scope for explicitly addressing *upstream* determinants (such as poverty, stigma, prejudice, discrimination) that may be serving to systematically limit individuals’ opportunities to enjoy high levels of SWB. The CA was developed in the 1980s by Amartya Sen in an attempt to transform welfare economics. Importantly, the CA moves away from approaches that prioritise the efficiency with which a goal can be achieved (i.e. the ‘means’) over consideration of the goal itself (i.e. the ‘end’). Instead of placing emphasis on utilities (i.e. access to resources such as income or assets), the CA focuses on promoting ‘the freedom that a person actually has to do things – things that he or she may value doing or being’ [28] (p.232). In the context of GMH initiatives, the CA provides a framework for endorsing processes that enable a person to be free to do the things that he or she may value doing or being. This can include accessing medical treatment, but also forms of social, educational, economic and political support that can help individuals and communities to flourish.

The following concepts are proposed to be important for articulating the CA:

1. Freedom. This is described as having two key aspects:
  A. Agency: A process aspect relating to the ability of an individual to act on behalf of what matters to him/her.
  B. Capability: A person’s potential to achieve valuable functionings from various good opportunities.
2. Functionings: Forms of valuable being and doing e.g. healthy body, being safe, being educated, having a good job.

The CA places particular emphasis on ‘agency freedom’; the ability to act in accordance with one’s ‘chosen goals and values as an element of a person’s effective power’ [29] (p.289). There is an important distinction between capabilities and functionings: *Capabilities* are understood to be the dimensions of SWB that are potentially available to the individual, whereas the *functionings* are the realized dimensions of SWB [30]. Another key distinction is the difference between ‘capacities’ and ‘capabilities’. Whereas the former are concerned with enhancing skills and abilities, the latter are regarded as a concept that incorporates the idea of increased choice and preparing people to grasp opportunities [31]. The CA places emphasis on the need to explore systemic socioeconomic and political barriers that curtail individual’s freedom - these are referred to as sources of ‘unfreedom’. The CA informed the development of the *Human Development Index* (HDI) [32] that is used by the United Nations to measure development.

The CA provides scope for policy initiatives to move beyond standardized, individualistic conceptualisations of ‘living well’ to a broader consideration of what it means for communities to be able to ‘live well together’ [33]. This is likely to be a particularly important consideration for understanding SWB in more collectivist contexts. It has been suggested that: ‘human wellbeing must be comprehended as being socially and psychologically co-constituted; where psychological processes engage with socially generated meanings to create the bridge between the individual human and social order’ [33] (p.510). By embracing a *social conception of wellbeing*, the CA can comprehend and manage the conflicts that might arise when one individual’s attempts to realise their freedoms are considered in the wider context of other people pursuing freedoms of their own (*ibid*). The ability to live together in social collectives is highlighted as being largely dependent on people reaching accommodation regarding each other’s systems of value and meaning. To help manage this process of accommodation, institutional procedures will be important for negotiating between parties about mutually beneficial outcomes (*ibid*).

This paper seeks to highlight a hitherto under-recognised source of inequity in the efforts being made to scale up mental health services across the globe i.e. a lack of parity in efforts to optimise people’s SWB vis-à-vis the focus allocated to treating symptoms of mental illness. The CA as a *human development* approach is highlighted as a framework for promoting greater equity in efforts being made across the globe to treat mental illness and optimise SWB. It is important to point out that our proposal is not intended to undermine efforts being made to treat mental illness, nor disregard research findings that have evaluated the efficacy of these efforts. Rather, it is an attempt to extend the remit of mental health initiatives to include a specific focus on supporting individuals and communities to have the freedom and capability to engage in valued functionings, which will provide important opportunities for enhancing SWB.

In particular, we propose that the CA could play an important role in guiding GMH initiatives that are seeking to build capacity for mental health services in LMIC where the vast majority of the global population live. Interventions aimed at promoting SWB may not need to be expensive, and the possibility exists that forms of support that people living in LMIC are already able to access may be helpful in this regard.

### The Capabilities Approach and social inclusion

Martha Nussbaum extended the work of Armatya Sen by exploring further the implications that the CA has for the realms of justice, social inclusion and citizenship [34]. She highlighted the strong moral and ethical credentials of the CA, with emphasis being placed on facilitating social contexts that promote and secure dignity for all members of society. Indeed, it has been suggested that social opportunities are crucial for expanding ‘the realm of human agency and freedom, both as an end in itself and as a means of further expansion of freedom’ [35] (p.6).

Nussbaum’s major contribution has been to emphasise that the CA can be experientially concerned with personal ‘narrative’ so as ‘to better understand people’s hopes, desires, aspirations, motivations and decisions’ [36] (p.104). In essence, the CA provides scope for reshaping the identities of individuals who have experienced mental health difficulties by providing opportunities to re-connect with society. Research investigating the narrative accounts provided by people experiencing mental health difficulties has indicated that these discourses can be characterized by experiences of exclusion [37]. The CA places emphasis on identifying sources of unfreedom such as discrimination and stigma. Disabling barriers that are synonymous with a sense of ‘patienthood’ are addressed, whilst simultaneously the individual is helped to realize their potential and enrich their sense of ‘personhood’ [38]. The inextricable link between ‘freedom’ and mental health had been highlighted before emergence of the CA. Dumont [39] observed that ‘the purposes of psychotherapy and social change are to widen the range of possibilities, to increase the option of human behavior. In short, to enhance freedom [....] The freedom I write about is not unrestricted individual initiative but the shared aspiration for the widest range of possibilities for all men. I call this aspiration mental health’ (p.50–51). The CA regards the removal of sources unfreedom as being necessary, but not sufficient, to enable human development – there is also a need to foster opportunities for people to engage with what they regard as valuable ways of being and doing e.g. the development of new or existing skills and opportunities to exercise these skills.

A number of researchers and theorists have applied the CA to the mental health arena, with the CA being highlighted as a way of facilitating people with a lived experience of mental health difficulties to engage with their values and priorities [40]. For example, the CA influenced the design and delivery of a community development approach to mental health promotion in a disadvantaged community in Sydney, Australia [41]. Specifically, three ‘action components’ were utilised that focused on developing: 1) Individuals and groups in the community (i.e. *people*); 2) Physical environments (i.e. *spaces*), and 3) The socio-cultural and historical characteristics of the communities (i.e. *places*) (*ibid*). Initiatives included an ‘employment action’ programme (including assistance with CVs, apprenticeships and training opportunities); the use of community graffiti walls; the cleaning up of local parks; the launch of a community garden, and long-term planning of social housing re-development all designed and delivered by the community (*ibid*). 

### Assessing capabilities

To facilitate the measurement of ‘capabilities’, a multi-dimensional instrument has been specifically developed and operationalized for use in mental health research in the UK i.e. the OxCAP-MH [42]. The capability domains covered by the instrument include: ‘health disability, meeting socially with friends, losing sleep over worry, enjoying recreational activities, having suitable accommodation, feeling safe, likelihood of discrimination, likelihood of assault (including sexual and domestic), ability to influence local decisions, freedom to express personal views, appreciation of nature, respecting and valuing people, enjoying friendship and support, self-determination, freedom of artistic expression and access to interesting activities or employment’ [43] (p.2). Preliminary research has indicated that the OxCAP-MH instrument demonstrated good acceptability and construct validity in a UK population [43]. Additional research should seek to facilitate the bottom-up development of measures aimed at assessing capabilities that are tailored to particular cultural contexts. It has been suggested that the aforementioned measure of IWB developed by White and colleagues for use in in Zambia and India captures the spirit of the CA because it aims to explore what people ‘think and feel themselves able to do and be’ [21] (p.268). Moving forward, it may be fruitful to conduct additional research aimed at developing similar types of measures in other settings.

At face value, measures such as the WHO Quality of Life Assessment [44] appear to be fairly consistent with conceptualizations of SWB. In developing the measure, the WHO defined QoL as: ‘individuals’ perceptions of their position in life in the context of the culture and value systems in which they live and in relation to their goals, expectations, standards and concerns’ [45] (p.1570). The position of the WHOQoL Group was that the WHOQoL poses questions about the ‘person’s global evaluations of behaviours, states and *capacities* and satisfaction/dissatisfaction with behaviours, states and *capacities* that inform about quality of life’ [44] (p.1405) (italics our own). As detailed earlier the distinction between ‘capacities’ and ‘capabilities’ is important here, with the latter focusing specifically on the freedom that individuals have to engage in behaviours that they *subjectively* value. So, although broadly compatible with the CA, the WHOQoL measure does not fully reflect the breadth of what the approach offers [46]. It has been argued that the CA provides a basis for a fuller and more dynamic view of QoL that focuses more on fulfilment (i.e. an individual’s subjective sense of developing to one’s full potential) as opposed to simply focusing on gratification (i.e. the short-lived indulging of a need/desire) [33].

### Global Mental Health and the Capabilities Approach

Moving forward, there is a real need for GMH as an area of study and practice to embrace a progressive and comprehensive framework that captures what it is seeking to deliver for the global population. As a human rights informed approach that highlights the importance of respecting democracy, voice, and diversity and enhancing social citizenship [38, 47], the CA offers great promise in guiding GMH-related initiatives. The need for GMH-related initiatives to be inclusive of the diversity of beliefs and practices espoused by individuals living in different parts of the world has been highlighted [48]. Consistent with these calls, consideration of human diversity is central to the CA. As Sen put it: ‘human diversity is no secondary complication (to be ignored, or to be introduced ‘later on’); it is a fundamental aspect of our interest’ [49] (p.11). According to Crocker and Robeyns [30], the CA takes account of human diversity in that it has an explicit focus on:

1. The plurality of particular functionings and capabilities as important evaluative spaces – it recognizes that these will vary within the individual.
2. The extent to which the personal factors (e.g. physical condition, intellectual ability etc.) and socio-environmental factors (e.g. public policies, social norms, geographical location, pollution etc.) permit an individual to convert a resource into valuable functionings.

As such, we are proposing the CA as a unifying framework for guiding Global Mental Health initiatives that can provide scope for implementing a diverse range of potential processes that are shaped by the particular contexts in which these processes are being applied.

### Addressing sources of ‘unfreedom’ and structural violence impacting on mental health and wellbeing

The *Mental Health Action Plan 2013–2020* [14] stated that: ‘Determinants of mental health and mental disorders include…social, cultural, economic, political and environmental factors such as national policies, social protection, living standards, working conditions, and community social supports’ (p.7). It is widely recognized that national mental health policies have not adequately extended beyond the health system, with insufficient focus being given to related sectors such as housing, education, social care, criminal justice and employment [50]. Indeed, it has been suggested that governments ‘in low resource settings need increased awareness of mental health as a cross-cutting development issue and the range of sectors that have a role to play in promoting mental health, preventing mental illness, and removing barriers to the participation of those with mental disability in their communities’ [51] (p.622). From a CA perspective, social inequalities can be viewed as sources of unfreedom that deprive people of opportunities to develop their capabilities, and engage in valued functionings. As Martha Nussbaum pointed out: ‘all nations are ‘developing nations’, in that they contain problems of human development and struggles for a fully adequate quality of life and for minimal justice. All are currently failing at the aim of ensuring dignity and opportunity for each person’ [52] (p.16). Consistent with its origins as a welfare economics framework, the CA provides scope for recognizing the important role that efforts aimed at reducing social inequalities can have in the context of GMH. As such, the CA provides scope for addressing factors relevant to mental health and wellbeing that are specific to the individual (i.e. *micro*-level) as well as those operating at a societal level (i.e. *macro*-level).

Boardman and colleagues [53] observed that ‘we need to be aware of: the pathways into and out of poverty; the impact of poverty on service users and their experiences; the associated forms of exclusion; and the barriers to life chances (p.28). The work of the organization *BasicNeeds* (http://www.basicneeds.org) exemplifies the positive impact that interventions aimed at addressing poverty and promoting mental health can have in LMIC. *BasicNeeds* pioneered a ‘Development Model for Mental Health’ that the organization has now applied in a wide-range of LMIC [54] Amongst several key priorities, the model used by *BasicNeeds* places emphasis on creating livelihood opportunities for people, which can do much to ameliorate socio-economic hardship - a pervasive source of unfreedom. An uncontrolled open trial of the application of *BasicNeeds*’ Development Model in rural Kenya indicated that over a 2-year there was a significant increase in numbers of people accessing support, as well as improvements in mental health, level of productive employment/income generation, quality of life and overall functioning among people with complex mental illness [55]. We propose that the work of *BasicNeeds* is consistent with the CA because of the opportunities that the organization facilitates for human development.

The term ‘structural violence’ has been coined to capture the way in which social structures and/or institutions harm people by stifling the possibility of their needs being met [56]. Examples of structural violence include institutionally propagated forms of suffering such as racism and poverty [57]. It is claimed that there has been a tendency to *de-socialize* particular difficulties that people experience (e.g. substance abuse) such that these are seen as intrapersonal or intra-psychic difficulties rather than also being influenced by societal factors [58]. We proposed that the application of the CA fosters opportunities for GMH initiatives aimed at building capacity for mental health services across the globe to *re-socialize* mental health difficulties and address social inequalities and structural violence as forms of unfreedom that may be serving to prevent individuals from realizing their capabilities and engage in valued functionings. Particular programmes have sought to address sources of unfreedom experienced by people with mental health difficulties in LMIC. For example, in Indonesia a programme of grassroots activism called ‘Indonesia Bebas Pasung’ (Free from Pasung) has used education to address long held traditional beliefs that impinge on current human rights and mental health standards (http://rightnow.org.au/writing-cat/review/breaking-the-chain/). The term ‘pasung’ is used to refer to the chaining/shackling of people with mental health difficulties. This has historically been a widespread practice in Indonesia. Programmes such as ‘Indonesia Bebas Pasung’ and ‘Aceh Bebas Pasung’ have shown promise in reducing the practice of pasung [59]. The application of the CA to initiatives of this kind would mean that campaigns aimed at promoting freedom would be complemented by concerted efforts to assist individuals to engage in valued functionings. Consistent with the CA, we propose that initiatives aimed at jointly addressing sources of unfreedom and supporting individuals to engage in value-consistent behaviours are required to enhance human development and optimize SWB.

The CA also provides scope for the focus of initiatives aimed at promoting mental health and wellbeing to consider sources of ‘unfreedom’ that sit outside sectors traditionally linked with mental health. For example, there is growing awareness that factors such as climate change [60, 61] and a lack of food security [62, 63] pose significant issues for people’s mental health. It has been suggested that these issues present challenges to mental health professionals because the particular pathological processes involved remain unclear [64]. LMIC will be particularly vulnerable to the mental health related impact of climate change because of existing disadvantage in socioeconomic status and the reduced availability of services for mental health [60]. Commentators have pointed out that the ‘agency’ aspect of the CA ‘expands the horizons of concern beyond a person’s own wellbeing, to include concerns such as slowing climate change or helping others’ [65] (p.11), whilst also facilitating a human development perspective for addressing food security issues [66].

### Facilitating critical reflection on Global Mental Health

The CA has been shown to be effective for critically examining the content of policy initiatives and the worthiness of these efforts [33]. Specifically, it has been suggested that:

1. The CA argues that individuals’ quality of life should be the principal concern of policy. This can be contrasted with a growing tendency in applied social sciences to concentrate on the means that may be used to enhance quality of life as an end itself.
2. The CA holds that human freedom and the ability to make decisions that impact on an individual’s life are integral to human dignity. This addresses concerns regarding the tendency for policy initiatives to regard people as the objects of policies.
3. The primacy that the CA gives to ethical considerations can be contrasted with a tendency to obscure ethical issues that can arise when employing technocratic approaches to address particular issues.
4. Being an approach rather than theory, there is flexibility in how the CA can be applied to issues, and the way in which it can be employed to reframe relevant issues (*ibid*).

Building on these observations, we propose that applying the CA to mental health policy initiatives (including those linked to GMH) can help highlight important opportunities that may been missed for facilitating individuals and communities to engage in valued functionings and realize their capabilities. Specifically, there is a need to be wary about the risk that:

1. Initiatives may at times focus predominantly on particular *means* for building capacity for mental health (e.g. the ‘scaling up’ of services to treat mental illness), rather than having a clear sense of what the desired ‘end’ actually is (i.e. clearer conceptualisations of ‘good outcome’ that include a reduction in illness levels but also a broader focus on improved subjective wellbeing and human development).
2. Policy initiatives could lapse into treating humans as the objects of policies and thereby impact adversely on human dignity.
3. Initiatives aimed at evaluating interventions for reducing purported ‘treatment gaps’ may have over-privileged technocratic approaches [e.g. methodologies prioritized by the Evidence Based Medicine (EBM) paradigm] whilst not adequately recognizing the important role that other forms of evidence might play (e.g. ethnography, qualitative research methods). Although EBM has many benefits, key limitations relating to the approach have been highlighted [67]. For example, evidence-based guidelines tend to map poorly onto complex multimorbidity, and statistically significant effects noted in clinical trails do not always equate to clinically significant change in practice. Specific concerns have also been raised about the way in which EBM approaches have been applied in the context of GMH e.g. the recruitment of unrepresentative samples and/or a publication bias skewed towards publishing significant findings only [68]. This has led to calls for the research agenda to be broadened to incorporate more trans-disciplinary approaches [68].
4. There is a risk that initiatives could be overly rigid in drawing heavily on Western models of treatment (e.g. allopathic medicine), whilst not giving adequate consideration to the benefits that non-allopathic approaches might have for treating mental illness and/or enhancing SWB [68]. It is claimed that policy initiatives and international networks may have served to undermine the perceived legitimacy and credibility of non-allopathic forms of support [69, 70]. This is in spite of research from Kerala, India where diverse forms of support (including allopathic medicine, Ayurveda, and religious healing) were all associated with improvements in individuals experiencing severe mental disorders [71].

### Applying the Capabilities Approach to Global Mental Health initiatives

Insights into the way in which the CA could potentially be applied in contexts relevant for GMH initiatives were provided in ethnographic research conducted with a community of Sudanese refugees/asylum-seekers in Cairo, Egypt [72]. The CA was highlighted as a framework for fostering a more sophisticated and deeply grounded understanding of community complexities that would be valuable for creating appropriate mental health services [72]. Consistent with this view, it has been suggested that the CA can serve as a ‘reasoning framework’ which: ‘recognizes universal principles and (at the same time) does not automatically devalue specific meanings and values found among different individuals both across different societies, and within a same society’ [33] (p.514). Clearly, there is a need to extend this work and explore the exciting possibilities that exist.

The CA approach provides a coherent framework that brings together the work of policy initiatives specifically aimed at mental health such as *mhGAP* [6, 7], *Social Determinants of Mental Health* [73], *Mental Health and Development: Targeting People with Mental Health Conditions as a Vulnerable Group* [74], and the *Comprehensive Mental Health Action Plan 2013–2020* [14], whilst also allowing space to incorporate other policy initiatives (e.g. those focusing on climate change and food security) that may limit people’s freedom to fulfil their potential and engage in valued functionings. As such, we propose that the CA represents a framework for supporting progress by complementing, consolidating and broadening the scope of existing initiatives, whilst also constructively guiding the direction of future initiatives aimed at promoting mental health and wellbeing. In being concerned with human development, the CA approach inherently facilitates opportunities to reduce levels of mental illness whilst also working to enhance mental wellbeing.

The application of the CA to Mental Health initiatives (including GMH) places specific emphasis on the need to systematically assess what individuals and communities value. ‘Values’ have been defined as: ‘learned, relatively enduring, emotionally charged, epistemologically grounded and represented moral conceptualizations that assist us in making judgments and in preparing us to act…Values can be grounded in the cultural heritage of a society and pervasively housed within the institutions of the society’ [75] (p.19). Particular forms of psychological intervention place a specific focus on helping individuals to clarify values. For example, *Acceptance and Commitment Therapy* (ACT) [76] aims to help individuals to explore their values, and engage in behaviours that are consistent with these values by enhancing psychological and behavioural flexibility. The aforementioned psychological intervention that has been found to be effective at promoting “flourishing” in individuals with depressive symptoms relative to waiting list control [20] used an ACT protocol. Research has indicated that psychological interventions can be adapted and implemented with positive outcomes in LMIC settings [77–79], importantly however there is a need to ensure that this is done in a culturally appropriate way and that valid outcome measures are used [80].

## Conclusions

This paper highlights an on-going lack of clarity about what constitutes a ‘good outcome’ for people who have experienced mental health difficulties. Specifically, a lack of equity was highlighted in efforts to reduce levels of mental illness compared with optimizing SWB. By highlighting human development as a key issue, the CA was identified as a framework for guiding initiatives across the globe that can focus on both optimizing SWB and reducing levels of mental illness. Furthermore, the CA provides scope for addressing both *micro-* and *macro-*level factors that may have served to thwart opportunities for individuals and communities to realize their capabilities. The application of the CA to GMH initiatives will serve to highlight the importance of developing a nuanced understanding about: 1) What individuals in particular settings value as being important to how they want to live their lives, and 2) The personal and structural factors that can promote, or hinder, individuals’ freedom to realise their capabilities and engage in valued functionings. Reprising the work of Vanessa Rose and colleagues (2012), we propose that the CA represents an important platform for considering factors related to people, spaces and places that can facilitate opportunities for enhancing mental health and wellbeing. We believe that this will provide opportunities for consolidating existing complementary mental health initiatives with a global focus, whilst also facilitating opportunities to extend into other non-health sectors. Importantly, the application of the CA can also provide opportunities to critically reflect on ways in which GMH initiatives could inadvertently hinder efforts to develop capabilities by: 1) Narrowly focusing on mental illness rather than also addressing subjective wellbeing, and 2) Relying too heavily on a restricted range of epistemologies and research methodologies to design and evaluate interventions.

## Abbreviations

CA: capabilities approach
GMH: Global Mental Health
LMIC: low and middle income countries
RA: recovery approach
